# Case Report: Molecular autopsy underlie COVID-19-associated sudden, unexplained child mortality

**DOI:** 10.3389/fimmu.2023.1121059

**Published:** 2023-04-18

**Authors:** Kana Unuma, Dan Tomomasa, Kosuke Noma, Kouhei Yamamoto, Taka-aki Matsuyama, Yohsuke Makino, Atsushi Hijikata, Shuheng Wen, Tsutomu Ogata, Nobuhiko Okamoto, Satoshi Okada, Kenichi Ohashi, Koichi Uemura, Hirokazu Kanegane

**Affiliations:** ^1^ Department of Forensic Medicine, Graduate School of Medical and Dental Sciences, Tokyo Medical and Dental University (TMDU), Tokyo, Japan; ^2^ Department of Pediatrics and Developmental Biology, Graduate School of Medical and Dental Sciences, Tokyo Medical and Dental University (TMDU), Tokyo, Japan; ^3^ Department of Pediatrics, Hiroshima University, Graduate School of Biomedical and Health Sciences, Hiroshima, Japan; ^4^ Department of Comprehensive Pathology, Graduate School of Medical and Dental Sciences, Tokyo Medical and Dental University (TMDU), Tokyo, Japan; ^5^ Department of Legal Medicine, Showa University School of Medicine, Tokyo, Japan; ^6^ Department of Forensic Medicine, The University of Tokyo, Tokyo, Japan; ^7^ Department of Life Sciences, Tokyo University of Pharmacy and Life Sciences, Hachioji, Tokyo, Japan; ^8^ Department of Pediatrics, Hamamatsu University School of Medicine, Hamamatsu, Japan; ^9^ Department of Medical Genetics, Osaka Women’s and Children’s Hospital, Izumi, Osaka, Japan; ^10^ Department of Child Health and Development, Graduate School of Medical and Dental Sciences, Tokyo Medical and Dental University (TMDU), Tokyo, Japan

**Keywords:** SARS-CoV-2, BCP-ALL, noonan syndrome (NS), sudden child death, autopsy

## Abstract

Herein, we report a child with COVID-19 and seemingly no underlying disease, who died suddenly. The autopsy revealed severe anemia and thrombocytopenia, splenomegaly, hypercytokinemia, and a rare ectopic congenital coronary origin. Immunohistochemical analysis demonstrated that the patient had acute lymphoblastic leukemia of the B-cell precursor phenotype (BCP-ALL). The complex cardiac and hematological abnormalities suggested the presence of an underlying disease; therefore, we performed whole-exome sequencing (WES). WES revealed a leucine-zipper-like transcription regulator 1 (*LZTR1*) variant, indicating Noonan syndrome (NS). Therefore, we concluded that the patient had underlying NS along with coronary artery malformation and that COVID-19 infection may have triggered the sudden cardiac death due to increased cardiac load caused by high fever and dehydration. In addition, multiple organ failure due to hypercytokinemia probably contributed to the patient’s death. This case would be of interest to pathologists and pediatricians because of the limited number of NS patients with *LZTR1* variants; the complex combination of an *LZTR1* variant, BCP-ALL, and COVID-19; and a rare pattern of the anomalous origin of the coronary artery. Thus, we highlight the significance of molecular autopsy and the application of WES with conventional diagnostic methods.

## 1 Introduction

Noonan syndrome (NS) is caused by pathogenic variants of genes encoding components of the RAS/MAPK signaling pathway (1), including *PTPN11*, *SOS1*, and *RAF1* (2). Recently, a leucine-zipper-like transcription regulator 1 (*LZTR1*) variant was found to be associated with NS using whole-exome sequencing (WES) (1). In 2021, it was reported that the prevalence rate of *LZTR1* variants in patients with NS was 4%–6% (2, 3), which was less than 50 cases (4).

The clinical characteristics of patients with NS harboring *LZTR1* variants are similar to those with other NS genotypes, including epicanthal folds, low-set ears, blepharoptosis, webbed neck, pectus excavatum-carinatum, cryptorchidism, short stature, intellectual disability, and cardiac anomalies (5). Meanwhile, abnormalities in stature, cardiac function, and neurodevelopment are considerably different between these groups of patients (6). For example, typical characteristics of children with NS include short stature related to growth hormone deficiency; however, only four cases of growth hormone deficiency have been reported in patients with NS harboring *LZTR1* variants (4).

Additionally, patients with NS typically present cardiovascular anomalies, with pulmonary stenosis, hypertrophic cardiomyopathy, and atrial septal defects—the most prevalent (3, 7). Correspondingly, 79.4% (27/34) of patients with NS harboring *LZTR1* variants reported in 2019 had heart disease, the most frequent being hypertrophic cardiomyopathy (71.4%) (2, 8). In a study of eight Japanese patients with NS harboring *LZTR1* variants, one patient (c. 742G>A, p.Gly248Arg, a variant in Kelch 4 domain) was diagnosed to have an anomalous origin of the coronary artery with peripheral pulmonary stenosis by echocardiography (8).

Patients with NS have an increased risk of hematological abnormalities (9–11); particularly, transient myeloproliferative disorder is observed in approximately 10% of pediatric patients (12). Juvenile myelomonocytic leukemia is another common hematological malignancy; approximately 90% of patients with NS and myelomonocytic leukemia have mutually exclusive *PTPN11, NRAS, KRAS, NF1*, or *CBL* variants (12). However, reports on association between *LZTR1* variants and hematological abnormalities are scarce.

Herein, we present a child who might have died due to COVID-19. A molecular autopsy of the patient revealed *LZTR1* variant-associated NS with a complex combination of acute lymphoblastic leukemia of the B-cell precursor (BCP-ALL) phenotype, and a rare ectopic congenital coronary origin.

## 2 Case history and symptoms at presentation

A 5-year-old boy, with no known medical history, developed a high fever (37.8°C) 6 days before his death. The patient was not administered any medication as the SARS-CoV-2 antigen test result was negative. On day 3 following fever onset, the patient’s body temperature dropped to below 37°C; however, his parents noticed that his face was abnormally pale. The fever (39.2°C) relapsed on day 5 after the initial episode. On day 6, the patient could barely drink or eat. When the patient’s mother tried to put him to bed, he moaned and convulsed with eyes open; the patient was rushed to the hospital emergency room *via* an ambulance. He went into cardiac arrest in the ambulance and died in the hospital despite the best efforts of cardiopulmonary resuscitation. The child’s blood analysis revealed severe anemia, thrombocytopenia, and hypercytokinemia, suggesting hemophagocytic lymphohistiocytosis (HLH). The detailed laboratory findings are summarized in **Table 1**.

**Table 1:** Representative laboratory findings of the patient’s blood analysis.

Test Name	Unit	Results	Normal Range
WBC	/μL	3.8 × 10^3^	3.3–8.6 × 10^3^
RBC	/μL	1.17 × 10^6^ ↓	4.35–5.55 × 10^6^
Hemoglobin	g/dL	3.3 ↓	13.7–16.8
Platelet	/μL	1.3 × 10^4^ ↓	15.8–34.8 × 10^4^
AST	IU/L	68 ↑	13–30
ALT	IU/L	20	10–42
LDH	IU/L	551 ↑	124–222
Albumin	g/dL	3.4 ↓	4.1–5.1
BUN	mg/dL	8	8–20
Creatinine	mg/dL	0.73	0.65–1.07
BNP	pg/mL	83.4 ↑	0–18.4
N-terminal pro BNP	pg/mL	2190 ↑	0–125
APTT	seconds	159.3 ↑	24–39
Fibrinogen	mg/dL	270	200–400
CRP	mg/dL	1.3 ↑	≤ 0.3
Interleukin-6	pg/mL	1470 ↑	≤ 7
Interleukin-18	pg/mL	5000≤ ↑	0–492

WBC, white blood cells; RBC, red blood cells; AST, aspartate aminotransferase; ALT, alanine aminotransferase; LDH, lactate dehydrogenase; BUN, blood urea nitrogen; BNP, B-type natriuretic peptide; APTT, activated partial thromboplastin time; CRP, C-reactive protein.(↑) means higher and (↓) means lower than reference values.

## 3 Autopsy findings

An autopsy was performed on the boy within 34 h after his death. Physical examination revealed the following: height, 108 cm; weight, 19.2 kg. No injuries were observed. The child was not prenatally diagnosed with any disease. The computed tomography scan showed no obvious acute or chronic fractures; furthermore, there were no major findings that pointed to a definite cause of death. The patient’s polymerase chain reaction test result was positive for SARS-CoV-2.

**Figure 1:**
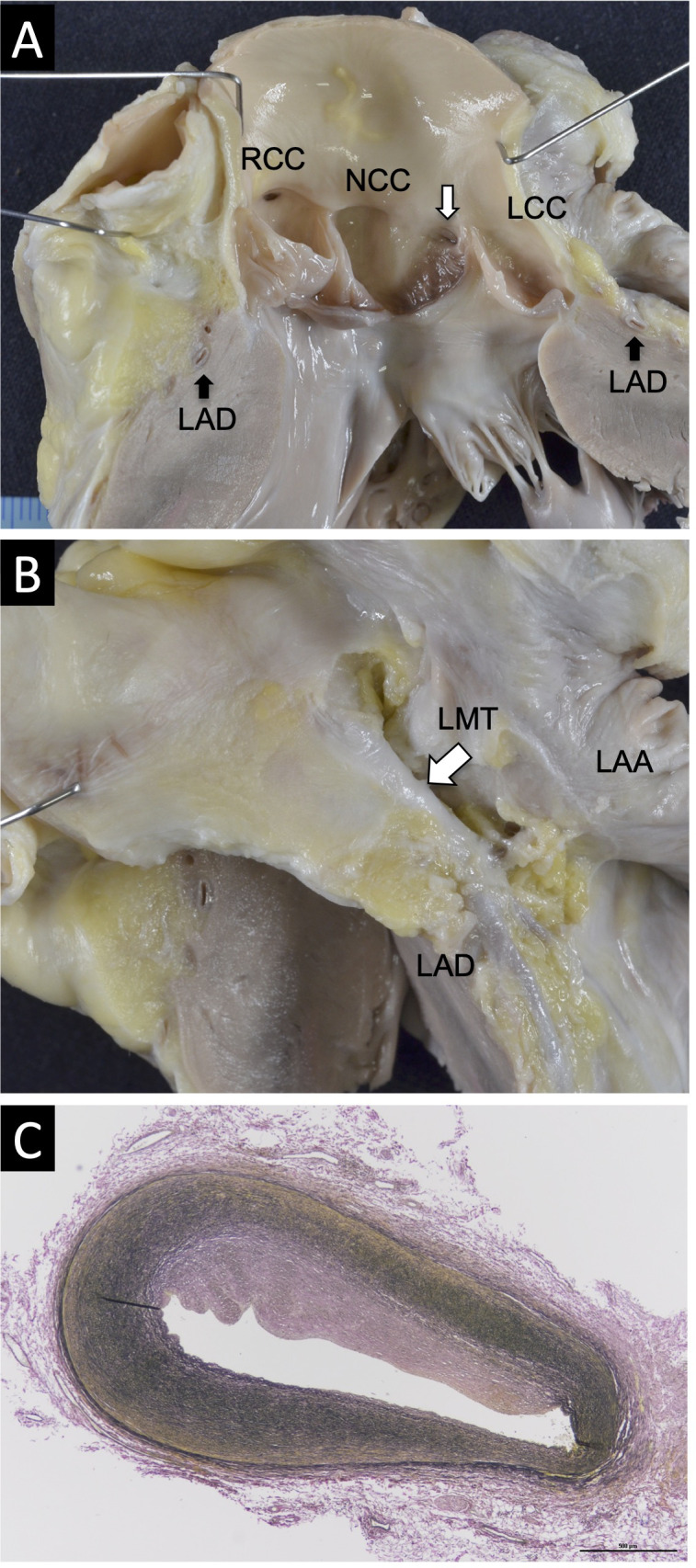
Macroscopic and microscopic pathological findings of the heart. **(A)** An acute angle take-off of the LCA from the NCC. **(B)** The LMT passing through a long course along the wall of Valsalva sinus. **(C)** Masson’s trichrome staining of the LMT revealed an eccentric intimal fibrous thickening indicating approximately 50% stenosis (magnification: ×10).

An autopsy revealed splenomegaly (140 g, normal range: 45–50 g) and hepatomegaly (810 g, normal range: 550–600 g). The heart weighed 122 g, which is within the normal range for a 5-year-old boy. We did not find an anomalous positioning, abnormal chamber arrangement, significant ventricular wall thickening, or chamber dilatation. However, an acute angle take-off of the left coronary artery (LCA) from the non-coronary cusp (NCC) was observed (**Figure 1A**). The left main trunk (LMT) passed through a long course along the Valsalva sinus wall (**Figure 1B**), and a histological section of the LMT revealed an eccentric intimal fibrous thickening indicative of approximately 50% stenosis (**Figure 1C**). No other macro/microscopic anomaly including COVID-19 pneumonia was found.

**Figure 2:**
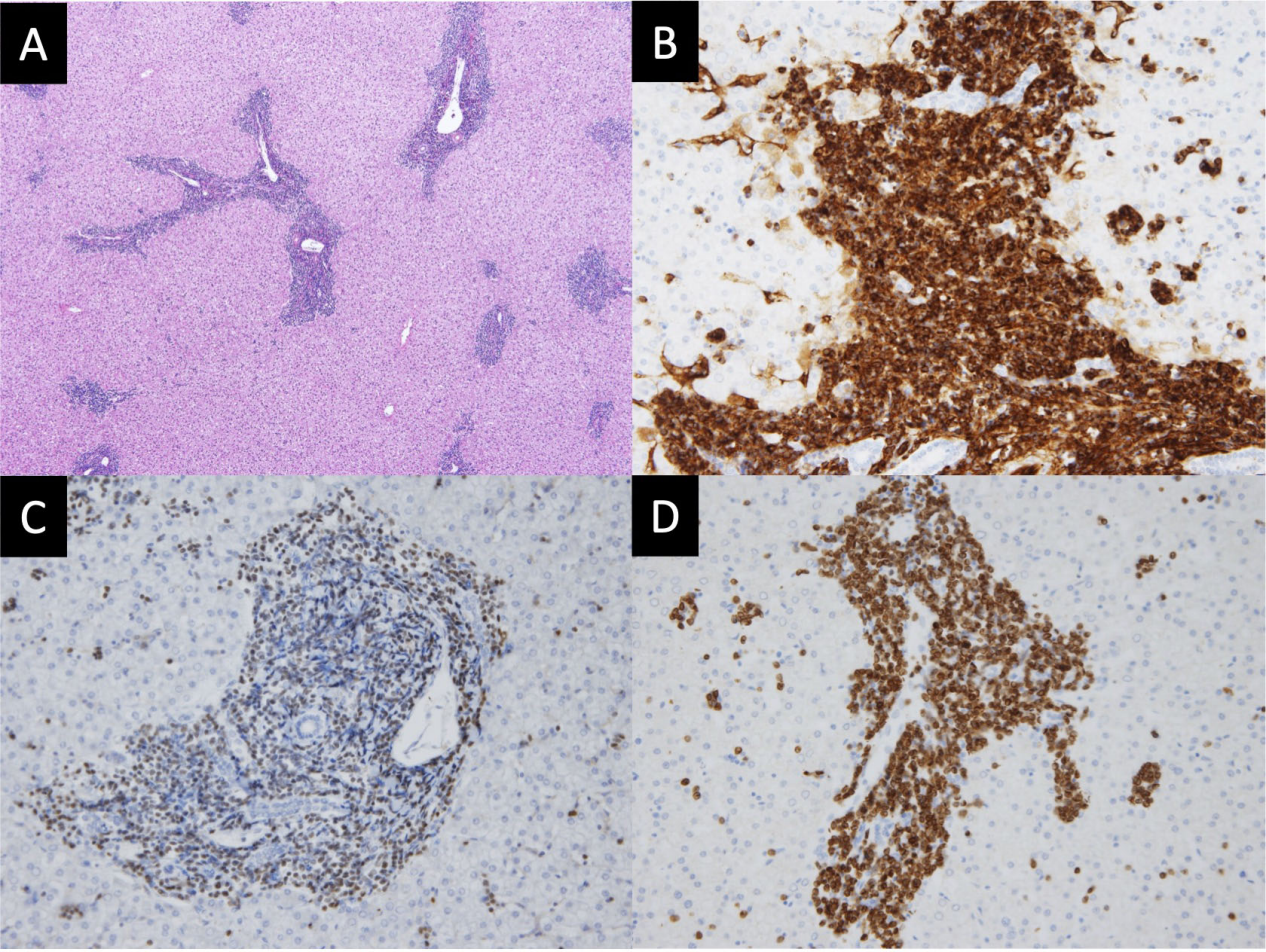
Histopathology and immunohistochemistry of the liver. **(A)** Hematoxylin and eosin (HE) staining depict diffused proliferation of lymphoblasts around Glisson’s capsule (magnification: ×200). **(B)** CD34 staining is positive in the cytoplasm and cytomembrane (magnification: ×400). **(C)** TdT staining is positive in the nucleus (magnification: ×400). **(D)** CD79a staining is positive in the cytoplasm and cytomembrane (magnification: ×400). These findings of the liver are consistent with the diagnosis of B-cell lymphoblastic lymphoma.

The child was diagnosed to have severe anemia and thrombocytopenia; therefore, further pathological examination was performed. Hematoxylin and eosin staining of the liver revealed diffused lymphoblast proliferation around the Glisson’s capsule (**Figure 2A**). Immunostaining of the liver using standard avidin–biotin immunohistochemical techniques showed positive staining of the cell cytoplasm and cytomembrane for CD34 (a hematopoietic stem cell marker; **Figure 2B**), TdT (a marker of precursor lymphoid cells containing B and T cells; **Figure 2C**), and CD79a (a pan-B-cell marker; **Figure 2D**). These findings were consistent with a pre-B-cell phenotype. The lymphoblasts also infiltrated the lung, spleen, kidney, and pancreas (**Supplementary Figure 1**).

HLH or the related inborn errors of immunity (IEI) were initially considered, and the child was screened for T-cell receptor excision circles (TRECs) and kappa-deleting recombination excision circles (KRECs). Moreover, tests for autoantibodies against type I interferon (IFN) were performed. The normal levels of TRECs and KRECs (965 and 817 copies/μg DNA, respectively) indicated no T-cell or B-cell immunodeficiencies. Moreover, the absence of autoantibodies against type I IFN suggested no COVID-19-associated IEI (13). The complex cardiac and hematological abnormalities suggested the presence of an underlying disease; therefore, we performed WES. Briefly, genomic DNA was fragmented using the Wizard^®^ Genomic DNA Purification Kit (Promega, Madison, WI, USA). Exonic sequences were enriched using xGen Exome Research Panel v2 (Integrated DNA Technologies, Coralville, IA, USA) and SureSelect XT HS Reagents (Agilent Technologies, Santa Clara, CA, USA). The captured fragments were purified and sequenced on DNBSEQ-G400RS (MGI Tech, Shenzhen, China) using paired-end reads. WES revealed a heterozygous *LZTR1* variant (c.1234C>T, p.Arg412Cys). Sanger sequencing revealed this variant in the nail, heart, brain, and spleen tissues, indicating that it is a germline variant (**Supplementary Figure 2**). Since the *LZTR1* variant is positive for PS1, PM2, PP2, and PP3, both *in silico* analysis and evaluation under the ACMG guideline indicate that it is likely pathogenic (**Supplementary Figure 3**). We have also analyzed the model structure of LZTR1 using AlphaFoldDB (**Supplementary Figure 4**). In the model structure, R412 is located on the loop of the Six-bladed beta-propeller domain, presumably forming an intermolecular interaction site, and its side chain forms hydrogen bonds with N410 and D86. Structural stability assessment of the R412C mutant using FoldX showed no significant change (-0.23 kcal/mol), suggesting that the mutation does not contribute significantly to structural stability (**Supplementary Figure 4**). However, it does alter the hydrogen bonding network of the loop structure, which may affect the molecular interactions. This variant has been reported in a few cases of NS (14, 15). Skilled geneticists identified mild facial features, such as broad forehead, blepharoptosis, epicanthal folds, hypertelorism, a short nose, and thick lips. However, no signs of a webbed neck or short stature were noted. Thus, the final diagnosis was NS associated with cryptorchidism, BCP-ALL, and a coronary malformation.

Additional blood analyses data are shown in **Table 1**. The results of split-surface general bacterial cultures of blood, cerebrospinal fluid, and lung were negative. The analysis results of throat swab fluid were negative for respiratory syncytial virus, adenovirus, and antigens of group A *Streptococcus*, influenza A, influenza B, and human metapneumovirus. Liquid chromatography–mass spectrometry revealed low caffeine concentrations in blood. No other drug was detected.

## 4 Discussion

In this study, the autopsy of the patient with COVID-19 revealed BCP-ALL and an anomalous origin of the LCA, and WES showed NS with an *LZTR1* variant. The severity of NS was relatively mild and, thus, NS was not suspected during the patient’s lifetime. Rapid cardiac arrest could have been caused by a COVID-19-related fever and fluid imbalance such as dehydration combined with coronary artery anomaly, which may cause fatal arrhythmias and relative ischemia. LCA arising from NCC, which was observed in our patient, is considered one of the rarest forms of coronary defects (16), detected in 0.02% (36 out of 174,262) of cases. Among the reported cases, 18 (50%) have been symptomatic, including 11 (31%) cases of sudden cardiac death.

Saji et al. (17) reported the death of a 13-year-old girl after long-distance running. Similar to our patient, this patient had LCA with marked intimal thickening. Other studies have also reported children who died suddenly after rigorous physical exercise, and the LCA of these patients also originated from the commissure between the NCC and left coronary cusp, with a slit-like orifice (18, 19). The origin of the LCA in these cases was similar to that observed in our patient, supporting our assumption of the mode of death described above.

Approximately 85% of childhood ALL cases are of B-cell precursor origin, whereas 15% originate from T cells (20). However, patients with NS frequently develop juvenile myelomonocytic leukemia and seldom ALL (21). Only a few cases of *LZTR1* variants in patients with NS and ALL are known. Chinton et al. (2) described a 2-year-old female NS/ALL patient with a variant (p. Gly248Arg) in the Kelch 4 domain. In a study of American patients with NS harboring *LZTR1* variants (n = 23), two (c. 2220-17C>A, p. Arg210* and c. 1678G>C, p. Glu563Gln) patients were identified to have ALL. One of the two patients developed ALL at 5 years of age, which progressed to acute myeloid leukemia at 7 years of age; the patient died 2 years later. The other patient developed ALL at 3 years of age and remained in remission (5). In addition, *Lztr1* deficiency has been linked to B-cell malignancies in CD19^+^B220^+^CD43^+^ immature B cells in mice (22). Thus, ALL development in our patient might be related to NS. However, the *LZTR1* variants associated with ALL are rare and need to be researched further.

Molecular autopsy refers to DNA-based identification of the cause of death. In recent large-scale studies of sudden death of young patients, molecular autopsies were able to uncover a likely or plausible cause of death in 12.6%–28% of cases (23, 24). Comprehensive molecular autopsy, similar to that performed on our patient, has the potential to provide more accurate information by identifying genetic causes of unexpected sudden death. Since WES-based molecular autopsy does not lie in the identification of variants but in determining their predicted pathogenicity, care must be taken not to erroneously determine ambiguous variants as pathogens (24). It should be noted that next-generation sequencing (NGS)-based target gene panel sequencing is useful for identifying the causative gene in a clinically suspected patient without accidental findings. However, WES can identify the causative gene even in patients without clinical diagnosis (25).

## 5 Conclusion

Herein, we presented a case of sudden child death. The death may have resulted from cardiac complications due to NS with a complex combination of BCP-ALL, COVID-19, and a rare pattern of an anomalous origin of the coronary artery. Our case study could be valuable for pathologists and pediatric practitioners as it emphasizes the significance of molecular autopsy. WES or whole-genome sequencing could be used in the diagnosis or even prevention of sudden child mortality.

## Data availability statement

The findings of the study are included in the article/**Supplementary Material**. Further inquiries can be directed to the corresponding author.

## Ethics statement

A forensic autopsy was performed on the boy as requested by the public prosecutor. For this type of case report, formal consent is not required. All procedures were performed in accordance with the ethical standards of our institutional research committee and tenets of the 1964 Helsinki Declaration and its later amendments.

## Author contributions

Conception and design of the research: KaU, HK. Acquisition of data: KaU, DT, KN, KY, TM. Analysis and interpretation of the data: KaU, TM, AH, YM, SW, TO, NO, SO, KO, KoU, HK. Writing of the manuscript: KaU, HK. Critical revision of the manuscript for intellectual content: TM, YM, HK. All authors contributed to the article and approved the submitted version.
