# Supplementary material for: Case Report: Molecular autopsy underlie COVID-19-associated sudden, unexplained child mortality
Source: Front Immunol. 2023 Apr 18;14:1121059. doi: 10.3389/fimmu.2023.1121059 (PMC10151512; doi:10.3389/fimmu.2023.1121059)
Supplement: Supplementary file 1 [file DataSheet_1.pdf]

Supplementary Material

Molecular Autopsy Underlie COVID-19-Associated Sudden, Unexplained Child Mortality

Kana Unuma*, Dan Tomomasa, Kosuke Noma, Kouhei Yamamoto, Taka-aki Matsuyama, Yohsuke Makino, Atsushi Hijikata, Shuheng Wen, Tsutomu Ogata, Nobuhiko Okamoto, Satoshi Okada, Kenichi Ohashi, Koichi Uemura, Hirokazu Kanegane

*** Correspondence:**

Kana Unuma

E-mail: unuma.legm@tmd.ac.jp

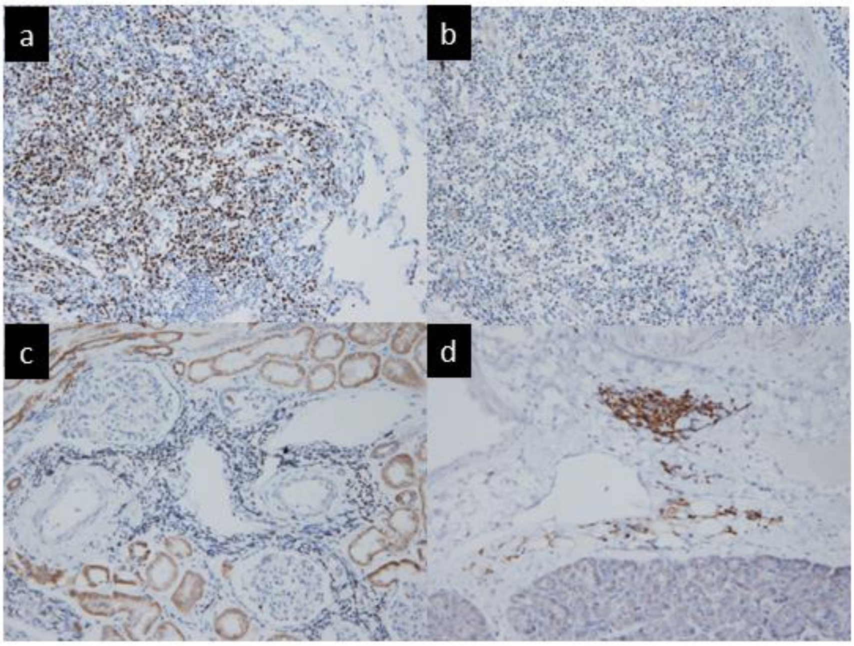

Supplementary Figure 1. Immunohistochemical staining of TdT. (**A–D**) The lymphoblasts infiltrated the lung (**A**), spleen (**B**), kidney (**C**), and pancreas (**D**). (×400).

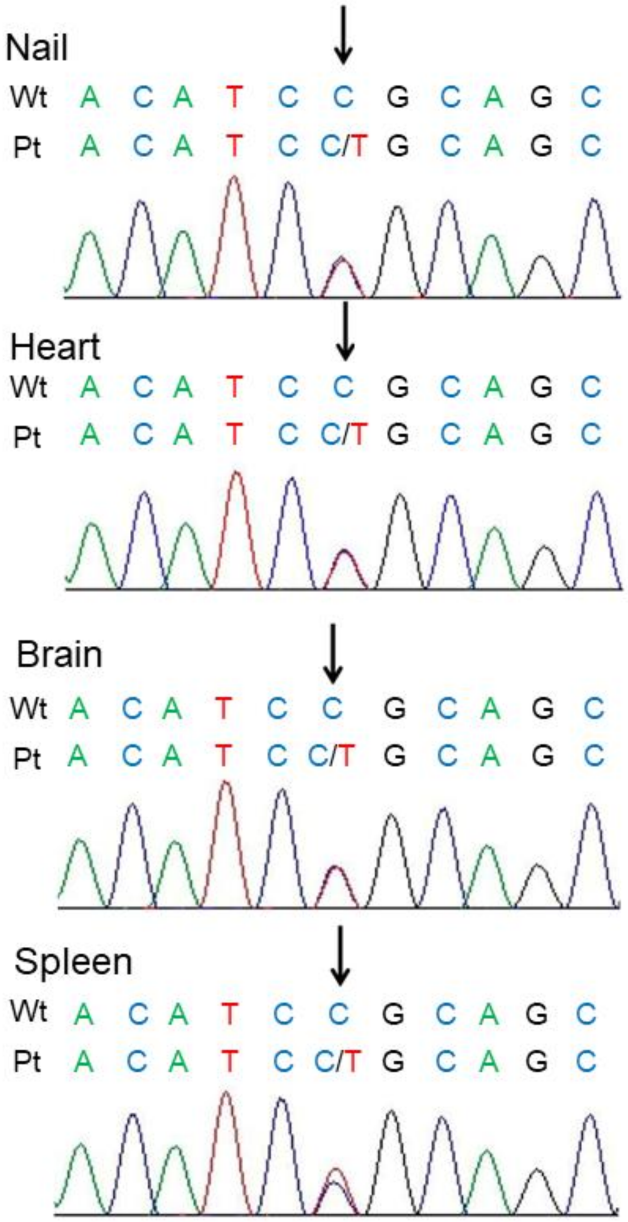

Supplementary Figure 2. Sanger sequencing of *LZTR*. Heterozygous variants (c.1234C>T) were identified in genomic DNA from the patient's nail, heart, brain, and spleen. Arrows indicate the position of c.1234C. Wt: wild-type, Pt: patient.

a

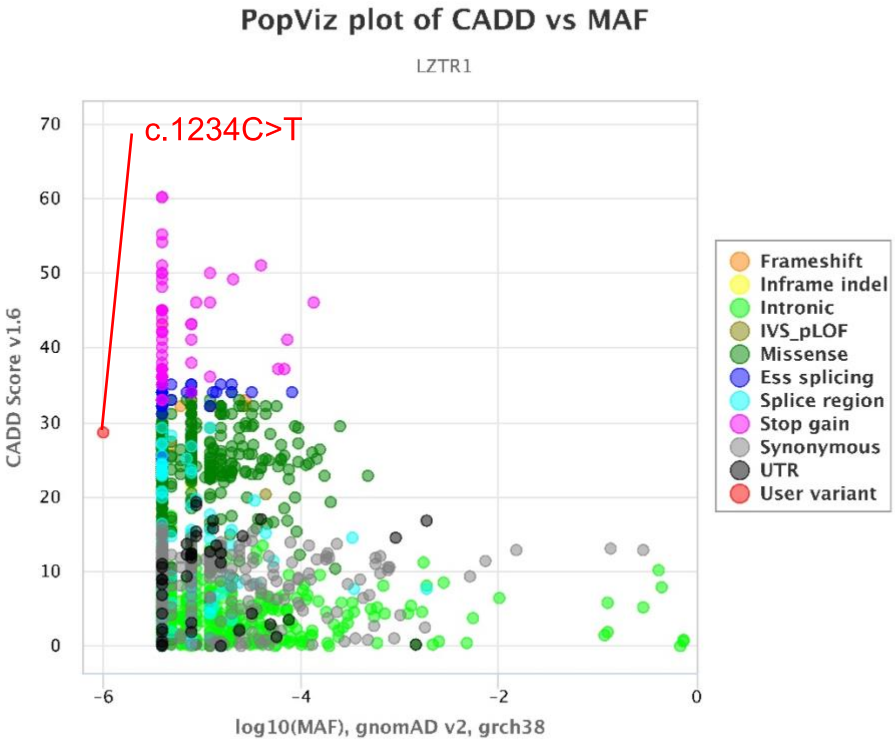

b

↓ (412)

species	…	410	411	412	413	414
H.sapiens		N	I	R	S	E
P.troglodytes		N	I	R	S	E
M.mulatta		N	I	R	S	E
C.lupus		N	I	R	S	E
B.taurus		N	I	R	S	E
M.musculus		N	I	R	S	E
R.norvegicus		N	I	R	S	E
G.gallus		N	I	R	S	E
D.rerio		N	V	R	S	E
D.melanogaster		S	V	R	R	D
A.gambiae		N	V	R	S	D
X.tropicalis		N	V	R	S	E

Supplementary Figure 3. *In silico* analysis of the *LZTR1* variant. (**a**) MAF and CADD scores of *LZTR1* by PopViz. The scores were -6 and 29 in the c.1234C>T variant, respectively. (**b**) The amino acid sequence alignment of *LZTR1* among species. The arrow indicates the missense variant (p.Arg412Cys) of the patient.

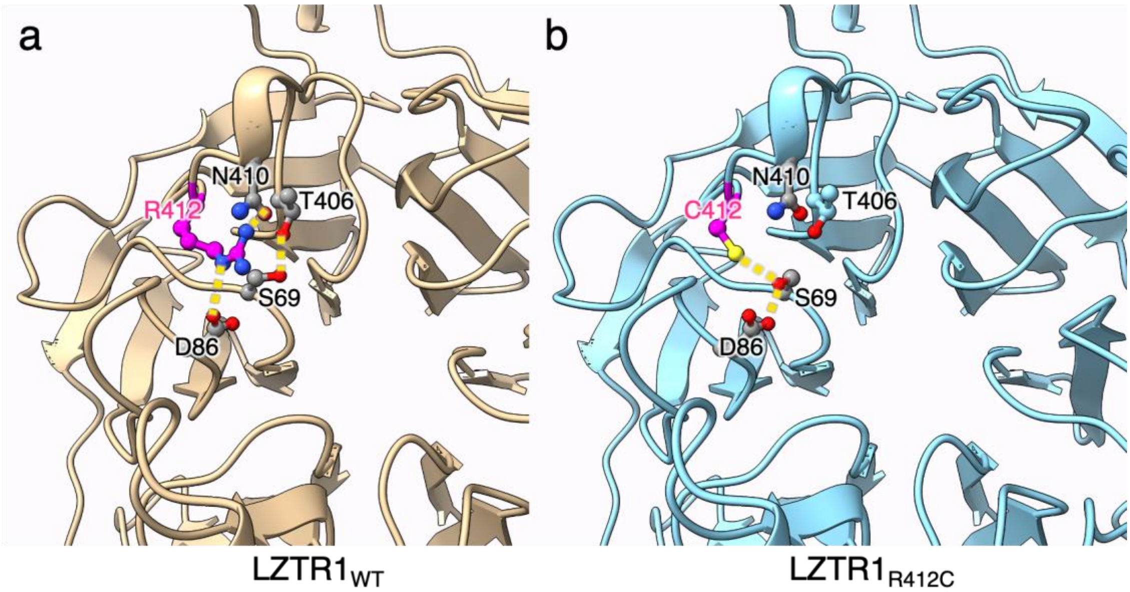

Supplementary Figure 4. Computational 3D modeling of LZTR1 variant using Alphafold. (a) Model structure of LZTR1 by AlphaFold2: see AlphaFold DB (https://alphafold.ebi.ac.uk/entry/Q8N653). (b) R412C mutant model structure generated by FoldX version 5 (https://foldxsuite.crg.eu/). Hydrogen bonding is indicated by yellow dotted line.
